# New Psychoactive Substances and Suicidality: A Systematic Review of the Current Literature

**DOI:** 10.3390/medicina57060580

**Published:** 2021-06-06

**Authors:** Stefania Chiappini, Alessio Mosca, Andrea Miuli, Maria Chiara Santovito, Laura Orsolini, John Martin Corkery, Amira Guirguis, Mauro Pettorruso, Giovanni Martinotti, Massimo Di Giannantonio, Fabrizio Schifano

**Affiliations:** 1 Psychopharmacology, Drug Misuse and Novel Psychoactive Substances Research Unit, School of Life and Medical Sciences, University of Hertfordshire, Hertfordshire AL10 9EU, UK; j.corkery@herts.ac.uk (J.M.C.); giovanni.martinotti@gmail.com (G.M.); f.schifano@herts.ac.uk (F.S.); 2 Department of Neuroscience, Imaging and Clinical Sciences, “G. D’Annunzio” University, 66100 Chieti, Italy; alessio.mosca909@gmail.com (A.M.); andreamiuli@live.it (A.M.); mariachiarasantovito@ymail.com (M.C.S.); mauro.pettorruso@hotmail.it (M.P.); digiannantonio@unich.it (M.D.G.); 3 Unit of Clinical Psychiatry, Department of Clinical Neurosciences/DIMSC, School of Medicine and Surgery, Polytechnic University of Marche, 60121 Ancona, Italy; laura.orsolini@hotmail.it; 4 Swansea University Medical School, Institute of Life Sciences 2, Swansea University, Swansea SA2 8QA, UK; amira.guirguis@swansea.ac.uk

**Keywords:** new psychoactive substances, NPS, suicide, suicidality, synthetic cannabinoids, synthetic cathinones, new synthetic opioids

## Abstract

*Background and Objectives*: Over the past twenty years a large number of new psychoactive substances (NPS) have entered and modified the recreational drug scene. Their intake has been associated with health-related risks, especially so for vulnerable populations such as people with severe mental illness, who might be at higher risk of suicidality or self-injurious behavior. This paper aims at providing an overview of NPS abuse and the effects on mental health and suicidality issues, by performing a literature review of the current related knowledge, thereby identifying those substances that, more than others, are linked to suicidal behaviors. *Materials and Methods*: A comprehensive and updated overview of the literature regarding suicidality and NPS categories has been undertaken. An electronic search was performed, including all papers published up to March 2021, using the following keywords “NPS” OR “new psychoactive substances” OR “novel psychoactive substances” OR “synthetic cannabinoids” OR “phenethylamines” OR “synthetic cathinones” OR “tryptamines” OR “piperazines” OR “new synthetic opioids” OR “designer benzodiazepines” AND (“suicide” OR “suicidality”) NOT review NOT animal on the PubMed, Cochrane Library, and Web of Science online databases. *Results*: Suicidality and self-injurious behavior appear to be frequently associated with some NPS such as cathinones, synthetic cannabinoids, and new synthetic opioids. The results are organized according to the substances recorded. *Conclusion*: The growing use of NPS has become a significant clinical issue, causing increasing concern and challenges for clinicians working in both mental health and emergency departments. Thus, considering the associations between NPS and suicidality or self-injurious behaviors, areas where suicide-prevention efforts and strategies might be focused are the early detection, monitoring, and restriction of NPS.

## 1. Introduction

The development and diffusion of new psychoactive substances (NPS) on the market has recently become a cause of serious concern [1]. In fact, in parallel with a decrease or stabilization in the use of internationally controlled drugs, the market for NPS continues to increase, with the Internet playing a pivotal role in contributing to this complex scenario [1]. The NPS market comprises a large number of substances, with new compounds being introduced continually [2]. These substances are drawn from a broad range of drug types and are not controlled by international drug laws. At the end of 2019, the European Monitoring Centre for Drugs and Drug Addiction (EMCDDA) was monitoring around 790 NPS, 53 of which had been reported for the first time in Europe in 2019, a number which represents a decrease compared with data previously recorded, reflecting the results of sustained efforts to restrict NPS production and control their diffusion [2]. Nonetheless, the number of NPS is vast, and includes the following categories: stimulants (e.g., cathinones, phenethylamines; tryptamines, etc.); synthetic cannabinoids; new benzodiazepines (e.g., etizolam, flualprazolam); synthetic opioids (e.g., fentanyl derivatives); hallucinogens (e.g., 1P-LSD and 4-AcO-DMT); and dissociatives [1,2,3]. Given their complex pharmacodynamics, there is an increasing level of concern about the onset of acute/chronic psychopathological consequences associated with NPS intake [1,3,4,5,6,7]. Moreover, the concurrent use of a range of different NPS, and/or medications, may be a reason for further clinical complications, including the emergence of substance-related psychotic phenomena [1,4,5,6,7,8]. Indeed, the consumption and frequent poly-consumption of NPS result in death, suicide, serious injury, and adverse effects on health [9]. 

Suicide is among the top twenty leading causes of death worldwide, with more deaths due to suicide than malaria, breast cancer, or war and homicide. Interestingly, suicide is the second leading cause of death in young people aged 15–29 years for both sexes, after road injury [10]. The risk of suicide in patients with psychiatric disorders is 5–15 times higher than in the general population [11,12,13]. In addition, substance use was found to be an independent risk factor for suicide attempt [14]. Substance use disorder (SUD) is considered an important risk factor for suicide, with vulnerable categories identified as younger age, history of psychiatric care, and opioid and alcohol use [15,16,17]. Indeed, substance use, substance intoxication, and pathological substance use have been demonstrated to be positively associated with suicidal behavior [18]. Moreover, neurobiological alterations, such as dopamine transporter availability in the basal ganglia, might be correlated to clinical presentations and psychopathological issues, including hopelessness, anhedonia, and dissociation, which may lead to suicidal thoughts, attempts, and actions [19].

During the Covid-19 pandemic, concerns about mental health and substance use have grown, including concerns about suicidal ideation. In a survey from June 2020, 13% of adults reported new or increased substance use due to coronavirus-related stress, and 11% of adults reported thoughts of suicide in the past 30 days [20]. Suicide rates have long been on the rise and may worsen due to the pandemic. Early 2020 data show that drug overdose deaths were particularly pronounced from March to May 2020, coinciding with the start of pandemic-related lockdowns. Several reasons, such as anxiety, fear of contagion, uncertainty, social isolation, chronic stress, economic difficulties, and other psychosocial issues related to the CoViD-19 pandemic have been leading to a relapse or exacerbation of pre-existing dual disorders and the onset of new dual disorders, thus increasing suicidality [21]. 

Aim of the study: The main outcome of this review was to investigate any correlation between the use of NPS and suicidality, performing a literature review of the current related knowledge, in order to understand if NPS abuse might be related with suicidal ideation and behavior, which are the most involved NPS, and identify categories of the users involved.

## 2. Materials and Methods

### 2.1. Systematic Literature Review Procedures

A systematic electronic search was performed on the 13 September 2020 on the following scientific search engines: PubMed, Scopus, and Web of Science (WoS). The following search strategies were used, respectively, in PubMed: (“NPS” OR “new psychoactive substances” OR “novel psychoactive substances” OR “synthetic cannabinoids” OR “phenethylamines” OR “synthetic cathinones” OR “tryptamines” OR “piperazines” OR “new synthetic opioids” OR “designer benzodiazepines”) AND (“suicide” OR “suicidality”) NOT review NOT animal; in Scopus: (TITLE-ABS-KEY (nps) OR TITLE-ABS-KEY (new AND psychoactive AND substances) OR TITLE-ABS-KEY (novel AND psychoactive AND substances) OR TITLE-ABS-KEY (synthetic AND cannabinoids) OR TITLE-ABS-KEY (phenethylamines) OR TITLE-ABS-KEY (synthetic AND cathinones) OR TITLE-ABS-KEY (tryptamines) OR TITLE-ABS-KEY (piperazines) OR TITLE-ABS-KEY (new AND synthetic AND opioids) OR TITLE-ABS-KEY (designer AND benzodiazepines) AND TITLE-ABS-KEY (suicide) OR TITLE-ABS-KEY (suicidality) AND NOT TITLE-ABS-KEY (review) AND NOT TITLE-ABS-KEY (animal)); and WoS: ((“NPS” OR “new psychoactive substances” OR “novel psychoactive substances” OR “synthetic cannabinoids” OR “phenethylamines” OR “synthetic cathinones” OR “tryptamines” OR “piperazines” OR “new synthetic opioids” OR “designer benzodiazepines”) AND (“suicide” OR “suicidality”) NOT review NOT animal). The systematic review was structured in accordance with the PRISMA [22] and PROSPERO guidelines [23]. Identified studies were assessed at title/abstract and full text screening against eligibility criteria. 

### 2.2. Data Synthesis Strategy

The searching of results was carried out individually by three investigators (A.Mi., A.M., and M.C.S.) and supervised by S.C. and M.P., doubtful cases were discussed with the professors G.M., M.D.G. and F.S. The selection and eligibility phase of the articles was carried out independently by the three members selected and then subjected to a final cross-check. Any doubts not solved by the team on the understanding of the topic covered in the article were requested directly from the author, if contactable. The data were collected in a Word table containing the first author’s name and year of publication of the study, study design, demographic variables (gender, age, psychiatric history), details on NPS taken (dosage, route of administration) and any other substances in combination, effects on suicidal behaviours, and suicidal ideation or abuse in order to commit the act/attempt. The data synthesis was carried out independently by two team members (A.M. and M.C.S.) and compared at the end of the extraction process.

The exclusion criteria for both selection phases were: (1) non-original research (e.g., review, commentary, editorial, book chapter); (2) non full-text articles (e.g., meeting abstract); (3) language other than English; (4) animal/in vitro studies; (5) articles not dealing with misuse of selected NPS (cannabinoids, phenethylamines, cathinones); (6) articles not dealing with suicide/suicidality; and (7) articles not dealing with substances consumed for the purpose of committing suicide. 

**Figure 1:**
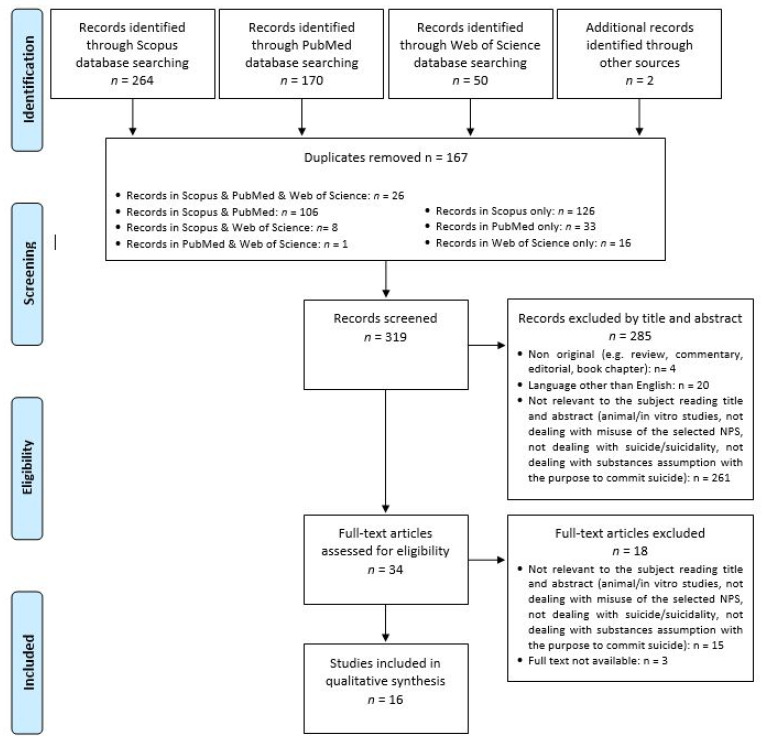
Flow-chart of study search and selection process according to PRISMA guidelines.

Removing duplicate articles (*n* = 167), from a total of 486 papers (PubMed = 170; Scopus = 264; WoS = 50; other sources = 2), a total of 319 records were screened, and, among these, 261 were irrelevant to the subject after reading the title and abstract (animal/in vitro studies, not dealing with NPS misuse or with serotonin syndrome), 20 were not written in English, and four were non-original articles (e.g., review, metanalysis, commentary, letter to the editor without data available, book chapter). Of the 34 full-text articles assessed for eligibility, 15 did not match the inclusion criteria for our review, and three were unavailable. Finally, 16 articles were taken into consideration for analysis (the operational method is illustrated in Figure 1). 

All the processes were conducted individually by A.Mi., A.M., and M.C.S., creating an Excel database. For dubious or missing results, the authors of the articles were contacted directly. All these research methods were approved by PROSPERO (identification code CRD42021234217).

For the purposes of this review, suicidal ideation refers to any thoughts of death, intention to kill oneself, or plan to end one’s life. Non-fatal suicidal behavior is understood as intentional self-injurious behaviour that is non-habitual and with a non-fatal outcome, while suicide refers to the act of deliberately killing oneself and is synonymous with fatal suicidal behavior [10]. 

## 3. Results

Sixteen eligible articles were finally identified and included in this systematic review. All results are summarized in Table 1.

The studies retrieved included: six case reports [24,25,26,27,28,29,30]; eight retrospective studies [31,32,33,34,35,36,37,38]; one cohort study [39]; and one case-control study [40]. Data mostly came from European countries, e.g., Finland [36]; Poland [26,28]; Slovenia [25]; Spain [37], and the United Kingdom (UK) [24,33]; but also from Australia [31,32]; Japan [34,35]; Turkey [39,40]; and the United States (US) [27]. Most cases involved young males (total M/F = 1837/223 = 8.23). NPS identified included the following categories: synthetic cathinones, e.g., 4-methyl methcathinone (4-MMC or mephedrone), 3- methyl methcathinone (3-MMC), 3,4-methylenedioxypyrovalerone (MDPV), alpha-pyrrolidinohexiophenone, alpha-pyrrolidinopentiophenone, 4- 4-chloromethcathinone and 4-fluoromethcathinone (flephedrone) [7,26,30,31,33,34,35,36,37,38]; synthetic cannabinoids, e.g., AB-CHMINACA, AB-FUBINACA, and JWH-018 [32,33,34,35,37,39,40]; phenetylamines, e.g., the β-keto-N-methylbenzodioxolylbutanamine (βk-MBDB) [24,28,31,33,37]; tryptamines [33]; piperazines [33]; aminoindanes [33]; the ketamine analogue methoxethamine [34]; synthetic opioids, e.g., acetyl fentanyl [35]; and a mix of synthetic cannabinoids and synthetic cathinones [25] or mix of drugs in general [37]. The most common route of administration, when indicated, was oral [24,25,26,28]; in one case the substance was smoked [27] and in one inhaled [30]. The dose was reported in one case only [28]. Psychiatric comorbidities, including a mood/anxiety disorder [25,30,39], a psychotic disorder [27,30], history of attempted suicide [24], or a SUD [25,27,40], were reported. Concomitant drugs used with NPS were benzodiazepines [25]; alcohol [24]; cannabis [30]; other NPS or a mix of other traditional licit drugs, or other prescription drugs, e.g., benzodiazepines, opioids, antidepressants, antipsychotics [31,32,33,34,35,36,38]; in the case of suicide by self-poisoning several drugs were detected in the post-mortem toxicological urine screening, including opiates/opioids, cocaine, amphetamines, benzodiazepines, antidepressants, antihistamines, mood stabilizers, Z-drugs, and antipsychotics, as well as cardiac drugs from the beta-blocker group and painkillers [26]. Finally, in several cases the outcome was fatal [24,26,28,31,32,33,36]. When reported, severe fatal self-poisonings [26,28,39], defenestration [24,33], hanging [33,39], burning [39], and firearms [33,39] were described. 

## 4. Discussion

To the best of our understanding, the current data represent the first systematic review of cases of suicide/suicide attempt involving NPS reported in the literature. Overall, the most represented NPS presenting an associated with these cases were the synthetic cathinones (e.g., 4-MMC, 3-MMC, MDPV, bk-MBDB. flephedrone, alpha-pyrrolidinohexiophenone, alpha-pyrrolidinopentiophenone, and 4-chloromethcathinone) and cannabinoids (e.g., AB-CHMINACA, AB-FUBINACA, and JWH-018). Both groups have been recognized as the largest categories of NPS identified in Europe last year [2] and were found in the increasing number of drug-related deaths recorded [41,42]. Cathinones are analogues of the naturally occurring cathinone found in khat (Catha edulis), and act as central nervous system stimulants related to the reuptake inhibition of noradrenaline, serotonin, and dopamine [1,5,7]. Cathinone intoxication might result in malignant serotonin syndrome, eventually causing a multi-organ dysfunction syndrome, coma, and consequently cardiac arrest and death [6]. Similarly to other stimulant NPS, such as phenethylamines [31], cathinone poisonings include psychiatric effects, e.g., psychomotor agitation, behaviour that is inadequate to reality, and even delusions and psychosis [1,5,7,43]. An increasing number of fatal poisonings, symptoms of addiction, and psychiatric disorders, including the risk of self-harm and suicide attempt, have been associated with the abuse of mephedrone, and cathinones in general, especially if they are combined with other psychoactive substances [36,44,45,46]. Consistent with data from previous studies [38], it is not clear whether the predominance of cathinones amongst cases of suicide reflects epidemiology or a propensity to induce suicidal behaviours. 

Synthetic cannabinoids have been shown to have significant medical and psychiatric adverse effects, including violent behavior, suicidal ideation, and self-harm amongst others [1,5,7,43]. Although the long-term risks of synthetic cannabinoids are still unclear, some studies suggest the possibility of inducing chronic psychotic symptoms and worsening underlying psychiatric illness [5,7,29]. The toxic effects of synthetic cannabinoids appear more severe and diverse than those associated with cannabis. In particular, synthetic cannabinoids exhibit cardiovascular and central nervous system effects more typically associated with psychostimulants. Synthetic cannabinoids have also been associated with delirium, psychosis, hallucinations, paranoia, and acute anxiety [1,5,7,43]. Death related to synthetic cannabinoid toxicity might be attributed to cardiovascular disease, agitated delirium, multiple organ failure, violent suicide, and traumatic accident [7,47,48,49,50,51,52,53]. As recorded by Kamijo et al. [34,35] consumption of NPS, specifically of synthetic cannabinoids and cathinones, can result in harmful behaviors, including violence to others or objects, traffic accidents, and self-injury or suicide attempts. Due the high prevalence of cathinones involved in hangings and other mechanical suicides (i.e., not suicide by drug overdose), a “cathinone phenomenon” has already been described [33,54]. Thus, considering the prevalence of synthetic cathinones and cannabinoids among the NPS here reported, most cases resulted in a fatal outcome. Overall, NPS use can result in severe and unpredictable consequences, which might be difficult to manage. Treatment of NPS intoxications, where recorded, was based on symptomatic and supportive care, because no specific antidote is available for synthetic cannabinoid and/or synthetic cathinone poisoning. All patients attempting suicide were admitted to emergency rooms or intensive care units. Oliveira et al. [27] presented a case of a suicide attempt by self-inflicted penetrating wound to the neck; the young man was brought to the emergency room after which he was transferred to the Psychiatry Department, exhibiting consistent improvement with his usual antipsychotic regimen. Unfortunately, according to the review findings, data on the users’ psychiatric diagnoses might have been underestimated or underrecognized, as they were not recorded. Interestingly, apart from cases associated with previous or ongoing psychopathological alterations, most cases appeared to be severe drug intoxications leading to disinhibition, impulsivity, loss of self-control, and alterations in judgment, thereby making suicide or suicidality more likely [37]. A case of schizophrenia was recorded [27]. Klavž et al. [25] reported a case of suicide attempt in a young man diagnosed with epilepsy, depression, drug dependence, and antisocial personality disorder. A history of suicide attempt [24,40] and of SUD were recorded in several cases [25,27,40], consistent with the literature identifying them as risk factors for suicide [10,18]. SUD has often been related to aggressive and suicidal behavior in previous research, and genetic contributing factors thought to be associated with suicide and aggression involve polymorphisms genes related to serotonin, norepinephrine, and dopamine systems, such as Catechol-O-methyltransferase (COMT; rs737865, rs6269, rs4633) [52]. 

Regarding worldwide data [10], globally the suicide rate is 1.8 times higher in males than in females, suggesting that men are at higher risk of substance use and that gender is an important variable in the etiology of suicidal behavior. Moreover, the specific role of gender in the association between substance use and suicidal behavior is complex and was not adequately investigated in the reviewed literature [10]. 

### Study Limitations

Despite the interesting data, the present review represents only a first assessment of data on NPS and suicidality, as provided by the current literature. The data may be influenced by publication bias, as studies that report negative or null associations often go unpublished. Furthermore, the use of an NPS might be underestimated, underrecognized, or complicated by difficulties in the analytical detection of NPS [55,56]. The majority of commonly employed designer drugs cannot be detected by routine hospital toxicological diagnostic management, especially in cases with an unclear and incomplete medical history. Therefore, physical examination of the patient becomes the basic tool in the diagnostic process [55,56]. Thus, considering that the possibility of identifying NPS in urine samples is complex and limited, a match between self-reported drug use and objective data is recommended, but might not always be considered reliable [37]. Another challenging limitation of this study is the difficulty/impossibility of establishing toxic/lethal concentrations, with there being an overlap between concentrations found in living and deceased individuals. In addition, due to various influences, the postmortem concentrations described should be regarded with reservation, and, due to the impossible implementation of systematic studies (e.g., controlled administration of NPS to living humans) for ethical reasons, no concentration–effect relationships can be established for the substances of interest. Moreover, often data extracted from post-mortem records exclude psychiatric diagnoses or concomitant (licit/illicit) drugs used. Finally, an interesting missing element is the sourcing of the NPS used (e.g., smart shops, Internet, etc.), which has not been reported here. Lastly, this review only included studies published in English.

## 5. Conclusions

Synthetic drugs constitute one of the most significant drug problems worldwide [2]. Consumption of NPS-containing products might cause severe health consequences and be involved in numerous drug-related deaths and suicides, as already described [57]. We have summarized here the current knowledge regarding NPS use and cases of suicide or suicide attempts. People with mental disorders, including SUDs, are at risk of suicide [10]; thus, early interventions in suicide prevention should include the identification of potential risk factors, such as psychiatric illnesses, SUDs, and the abuse of licit/illicit drugs and NPS, which must be explored, assessed, and addressed in the management plan of likely suicidal thoughts or behaviours. For these reasons, it is necessary to educate the scientific community, health care professionals, and drug users on the psychological and medical aspects of taking NPS, and especially combining them with additional substances. This can result in harmful effects and greater risks of psychopathological consequences, including not only hospitalization but suicide attempts. Public health policy, research, and clinical attention should focus on suicide prevention and reduction of the morbidity and mortality associated with suicidal behaviour.

## Figures and Tables

**Table 1:** Main findings of the retrieved studies.

Ref.	Population (*n* TOT)	Mean Age (YRS, SD)	Type of NPS	Psychiatric Comorbidity	Dosage and ROA	Poly-Abuse (substance)	Concomitant Drugs	Actions Taken and Outcome	Notes
CASE REPORTS									
**Carter et al., 2000** [24]	*n* = 1 (M)	33	N-methyl-1-(3,4-methylenedioxyphenyl)-2- butanamide (MBDB)	History of attempted suicide	ND dose; oral	Alcohol	None	Suicide (died falling from a height)	MBDB blood concentration of 1.2 mg/l
**Klavž et al., 2016** [25]	*n* = 1 (M)	38	Mix of synthetic cannabinoids and synthetic cathinones (AB-CHMINACA, AB-FUBINACA, Alpha-pyrrolidinohexiophenone, Alpha-pyrrolidinopentiophenone and 4- 4-chloromethcathinone)	Epilepsy; Depression; Drug dependence; Antisocial Personality Disorder	ND dose; oral	Benzodiazepines	Lamotrigine, Fluoxetine, Valproate, Diazepam, Zolpidem and Promazine	Suicide attempt	
**Margasińska-olejak et al., 2019** [26]	*n* = 1 (F)	19	3- methyl methcathinone (3-MMC)	ND	ND dose; probably oral	None	Opiates, Methadone, Cocaine, Amphetamines, Benzodiazepines, Antidepressants, Phenothiazine derivatives, Carbamazepine, Z-drugs, Haloperidol, Risperidone, as well as cardiac drugs from the beta-blocker group and painkillers	Suicide by self-poisoning	3-MMC blood concentration of 800 ng/ml
**Oliveira et al., 2017** [27]	*n* = 1 (M)	32	Mix of several synthetic cannabis analogues (‘Shiva Ultra Strong’)	Paranoid schizophrenia; history of drug abuse (alcohol and heroin); on treatment with Haloperidol Decanoate 100 mg/28 days; Clozapine 200 mg/day; and Lorazepam 2.5 mg day	ND dose; smoked	None	None	Suicide attempt by self-inflicted penetrating wound to the neck; brought to the emergency room after which he was transferred to the Psychiatry Department, exhibiting consistent improvement with his usual antipsychotic regimen	
**Rojek et al., 2012** [28]	*n* = 1 (M)	21	2-methylamino-1-(3,4-methylenedioxyphenyl) butan-1-one (bk-MBDB)	ND	10 tablets of unspecified dose; oral	None	None	Suicide attempt; after admitted to Intensive Care unit, he died from cardiac and respiratory arrest	The preparation was called ‘Amphibia’; serum concentration was found 20 mg/L
**Thomas et al.** [29]	*n* = 1 (M)	20	K2	ND	ND dose, smoked	None	None	Brought by police to the ED with acute agitation, confusion, suicidal ideation, and self-inflicted trauma after smoking. Once medically stabilized, he was transferred to the inpatient psychiatric unit for continued monitoring	
**Thornton et al., 2012** [30]	*n* = 1 (M)	23	3,4-methylenedioxypyrovalerone (MDPV), and 4-fluoromethcathinone (flephedrone)	He had a history of being prescribed Clonazepam, Quetiapine, Aripiprazole, Valproic acid, and Lithium	ND dose; inhaled	Cannabis	None	Arrived to the ED with bizarre behaviour, suicidality, and hallucinations. He was physically and chemically restrained. Agitation and psychosis solved after IV lorazepam (6 mg) and droperidol (2.4 mg)	MDPV serum concentrations was 186 ng/mL; flephedrone serum concentration was 346 ng/mL
RETROSPECTIVE STUDIES									
**Darke et al., 2019** [31]	*n* = 82 (M = 71) cases where new psychoactive stimulants were contributing to death were retrieved from the National Coronial Information System (2000–2017)	30,7 (SD = 10.4)	Cathinones or phenethylamines	ND	ND	ND	Psychostimulants (e.g., methamphetamine, MDMA, cocaine, dimethylamylamine); Opioids (e.g., morphine, methadone, fentanyl, buprenorphine, tramadol, oxycodone, hydromorphone); Alcohol; Cannabis; Synthetic cannabinoids; Hypnosedatives; Antidepressants; Antipsychotics	Unspecified suicide, *n* = 10 (M = 8)	Of the cases of suicide, 8 were positive for cathinones (methcathinone, MDPV and alpha-pyrrolidinopentiophenone) and two for phenethylamines
**Darke et al., 2020** [32]	*n* = 55 (M = 50) cases where synthetic cannabinoid use was a mechanism contributory to death were retrieved from the National Coronial Information System (2000–2017)	37,2 (SD = 12.0)	Unspecific synthetic cannabinoids (most commonly reported synthetic cannabinoids were AB-CHMINACA and JWH-018)	ND	ND	Other substances were present in 42 (76.4%) cases, including Alcohol (34.5%), Cannabis (23.6%), other NPS (cathinone, phenethylamine), and Phencyclidine	Antidepressants, Benzodiazepines, and Antipsychotics were each present in substantial minorities. Also, Psychostimulants (methamphetamine, MDMA, phentermine) and Opioids (morphine, methadone, buprenorphine, tramadol, oxycodone) were recorded	Unspecified suicide, *n* = 6 (M = 5)	
**Elliot and Evans, 2014** [33]	*n* = 203 NPS-related deaths detected post-mortem samples between January 2010 and December 2012 (17% were fatal hangings and 5% involved other manners of mechanical suicide, e.g., struck by a train, asphyxia, fatal gunshot wound or jump/fall)	ND	Cathinones (e.g., mephedrone, MDPV, 4-methylethcathinone) were involved in 41% of hangings or other mechanical suicides (i.e., not suicide by drug overdose); other NPS detected: Piperazine; Tryptamine; Phenethylamines; Aminoindans; Synthetic cannabinoids	ND	ND	ND	Paracetamol (13.4%), Citalopram (12.7%), Diazepam (8.4%), Mirtazapine (8.0%), Zopiclone (6.8%), and Cocaine (6.5%)	Suicide, *n* = 44	
**Kamijo et al., 2014** [34]	*n* = 518 (M = 425) patients who were transported to emergency facilities between January 2006 and December 2012 after consuming synthetic chemicals	28.4	Synthetic cannabinoids, synthetic cathinones, and methoxetamine	ND	Inhalation, ingestion, sniffing, inserted anally	ND	Alcohol, Benzodiazepines	Self-injury or suicide attempts were observed in four patients	
**Kamijo et al., 2016** [35]	*n* = 589 (M = 528) patients who were transported to emergency facilities after consuming NPS-containing products (January 2013-December 2014)	30	Synthetic cannabinoids (AB-CHMINACA); synthetic cathinones (α-PHP and 2-(ethyl amino)-1-(4-methylphenyl) pentan-1-one); acetyl-fentanyl	ND	Inhalation, ingestion, inserted anally	Barbiturates; Cannabinoids; Phencyclidine; Amphetamines; Opiates; Cocaine	Benzodiazepines, Antidepressants	Self-injury or suicide attempt, *n* = 6	
**Kriikku et al., 2015** [36]	*n* = 38 (M = 30) 3, 4-methylenedioxypyrovalerone (MDPV)-positive post-mortem cases	28.3	MDPV; other NPS were present in 24 % of the cases	ND	ND	ND	Amphetamines; Opioids; Alcohol; Benzodiazepines; Cannabis	Unspecified suicide, *n* = 9	MDPV blood concentration was 0.12 mg/L; victims in MDPV-positive suicides were significantly younger than those in other MDPV-positive fatalities
**Martinotti et al., 2021** [37]	*n* = 38 on 110 subjects admitted to the Can Misses Hospital’s psychiatry ward in Ibiza (2015–2019)	ND	Psychodepressors (e.g., opioids, alcohol, benzodiazepines), Psychostimulants (e.g., cocaine, amphetamines, synthetic cathinones); Psychodysleptics (e.g., cannabinoids, psychedelics, dissociatives)	ND	ND	Multiple substance use was recorded (77.7%)	ND	Suicide thoughts was evidenced in 35% (*n* = 38) of the sample as to the suicide item of the HM.A.-D, with 18% (*n* = 20) reporting a severe suicide risk. The assessment of suicidal risk at admission as to the C-SSRS was performed in 63 subjects of the total sample: 25 (39%) patients were positive for suicide attempts (*n* = 6), suicidal ideation (*n* = 9), or death ideation (*n* = 10)	Suicide Ideation Intensity overall and in the previous month was higher in users of opioids and in general of psychodepressors. Impulsivity and loss of self-control may be determinants of the increased suicidality irrespectively of any major ongoing psychiatric background
**Ordak et al., 2020** [38]	*n* = 601 (M = 559) patients addicted to mephedrone who were admitted to a psychiatric hospital between 2010 and 2018 due to regular mephedrone intake	26–35	Mephedrone	ND	ND	Opioids; Benzodiazepines; Alcohol; Cannabinoids	Opioids, Benzodiazepines	Suicide attempts, *n* = 147	Growing year-on year percentage of people who attempted suicide because of regular mephedrone intake. The more psychoactive substances were combined, the greater was the risk of attempted suicide
COHORT STUDIES									
**Oznur et al., 2018** [39]	*n* = 77 (M, performing compulsory military service)	22.38 (SD = 3.92)	Unspecified synthetic cannabinoids	Adjustment disorder; 49.4% of the sample had a history of suicide and 63.7% had a self-mutilation history	ND	ND	ND	16 out of 27 people who used synthetic cannabinoids attempted suicide (59.3%); also, 18 of 27 cases using synthetic cannabinoids (66.7%) had a history of suicide attempts. Of all the patients who attempted suicide, 83.1% (*n* = 64) selected methods unlikely to fail including firearms, hanging, jumping, cutting tools, and burning, while 16.9% (*n* = 13) chose a method with a greater chance of rescue (drug overdose)	There was a significant relationship between the use of synthetic cannabinoids and suicide attempts. No statistically significant relationship was found between the suicide attempt and other substances, except synthetic cannabinoids
CASE-CONTROL STUDIES									
**Pehlivan et al., 2020** [40]	*n* = 94 (M = 92)	28.03	Unspecified synthetic cannabinoids	SUD	ND	ND	ND	Suicide attempts, *n* = 19	The COMT variants were associated with self-mutilation (Val108Met) or attempted suicide (Val158Met) in patients with synthetic cannabinoids use disorder

C-SSRS: Columbia suicide severity rating scale; COMT: Catechol-O-methyltransferase; ED: emergency department; F: female; HM.A.-D: Hamilton depression scale; M: male; N/A: not applicable; NPS: new psychoactive substances; ROA: route of administration; SD: standard deviation; SUD: substance use disorder.

## Data Availability

Not applicable.
